# Successful discharge from a mental health halfway house: A personalized process with integrated approaches

**DOI:** 10.1192/j.eurpsy.2021.1349

**Published:** 2021-08-13

**Authors:** F.-P. Chen

**Affiliations:** Department Of Social Welfare, National Chung Cheng University, Chiayi County, Taiwan

**Keywords:** psychiatric rehabilitation, mental health halfway house, discharge

## Abstract

**Introduction:**

In Taiwan, residents of mental health halfway houses (MHHH) receive psychiatric rehabilitation services, aiming for independent living and community integration. Research is yet to investigate how MHHH may effectively assist residents’ discharge in this cultural context.

**Objectives:**

To examine the processes of assessment, preparation, assistance, and appraisal of discharge from MHHH staff’s perspectives.

**Methods:**

Semi-structured in-depth interviews were conducted with 11 halfway house staff members. Verbatim transcripts were analyzed with dimensional analysis procedures of the grounded theory methodology.

**Results:**

Successful discharge is a personalized process with integrated approaches addressing three essential factors: (a) regular community involvement, (b) the residents’ capacity to work, and (c) the family’s acceptance and support. Staff supported individual residents’ community involvement by attending to residents’ personal interests, resource availability, financial concerns, and transportation. Moreover, staff provided rehabilitation trainings to develop work capacity. However, residents’ motivation and functioning as well as job opportunities might affect their employment. Finally, in Taiwan, residents were rarely discharged without their family members’ consent because residents tended to co-reside with their family after discharge or rely on family support while living separately. Staff worked to engage families, which was influenced by family relationship quality, past traumatic events, financial concerns, capacity to assist the resident, and/or the resident’s ability to assist with family affairs.

**Conclusions:**

To achieve successful discharges, MHHH staff need to assist each resident by developing an integrated plan to enhance conditions of the aforementioned factors, including strategies for different familial situations to garner family support in this cultural context.

